# Prospective Study of 506 Dogs with Tick Paralysis: Investigating Measures of Severity and Clinical Signs as Predictors of Mortality and Assessing the Benefits of Different Therapeutics

**DOI:** 10.3390/ani14020188

**Published:** 2024-01-06

**Authors:** Rick Atwell, Dianne Vankan

**Affiliations:** School of Veterinary Science, The University of Queensland, Gatton 4343, Australia; r.atwell@uq.edu.au

**Keywords:** tick paralysis, tick antiserum, mortality rates, visual analogue scale, *Ixodes holocyclus*, facial expressions

## Abstract

**Simple Summary:**

Tick paralysis (TP) in dogs caused by *Ixodes holocyclus* can be perplexing for clinicians, with some dogs recovering rapidly after the administration of tick antiserum and others deteriorating before improving or dying despite clinical interventions. The prediction of individual case disease progression, recovery time, and mortality is difficult. This study collected a large body of clinical data from natural cases of TP across the eastern seaboard of Australia, with the veterinarians involved receiving training prior to the tick season to use two new tools to assess disease severity (visual analogue scales (VAS) and facial expressions of distress) as well as established methods of assessment. VAS scores for respiratory dysfunction and muscular weakness were highly predictive of mortality, but established methods for respiratory evaluation were not. Specific respiratory clinical signs present at admission and during hospitalisation were identified as highly predictive of mortality, and other specific clinical signs were shown to be highly predictive of prolonged hospitalisation times. These findings will assist in the management of TP cases: veterinarians can utilise the risk factors for mortality reported here to improve their communication with owners about prognosis and to make timely decisions to prioritise intensive care therapy.

**Abstract:**

Survey data from 42 Australian eastern seaboard veterinary practices involving 506 cases are reported with regard to clinical signs, disease severity, mortality, use of pharmaceuticals, and recovery times. New measures of disease severity (visual analogue scales (VAS) and facial expressions) were tested alongside “gold standard” measures (neuromuscular junction (NMJ) scores). Univariable and multivariable logistic regression analyses were conducted to evaluate associations between variables. The VAS scores were progressive, prognostic (especially the respiratory scores) and correlated with the NMJ scores. The presence of inspiratory dyspnoea and crackles on the day of hospitalisation, progressing to expiratory dyspnoea and an expiratory wheeze 24 h later, were highly predictive of mortality. Altered facial features on hospital admission were also highly predictive of mortality. The previously used respiratory score (using various clinical signs) was not predictive of mortality. Older animals had a higher mortality rate, and no gender or breed susceptibility was found. The only pharmaceuticals that were positively associated with mortality were tick antiserum and, in severe cases, antibiotics. The use of many pharmaceutical products (acepromazine, atropine, steroids, antihistamines, antiemetics, diuretics, and S8 anti-anxiety and sedation drugs) had no effect on mortality. More drug classes were used with increasing clinical severity and specific factors (e.g., vomiting/retching, hydration) affected the period of hospitalisation. Geographic variation in respiratory signs and toxicity scores was evident, whereas mortality and disease severity were not different across regions.

## 1. Introduction

Tick paralysis (TP) caused by *Ixodes holocyclus* defines the wide array of clinical presentations from paretic to non-paretic, involving, for example, cardiopulmonary dysfunction. TP has been studied in many ways over the last few decades. Research data have been generated in PhD studies covering neuromuscular junction (NMJ) pathophysiology [1]; physiological parameters in induced disease in Greyhounds [2]; cardiac dysfunction [3]; aspects of hypothermia and respiratory dysfunction [4]; and the tick antiserum (TAS) reaction [5]. Although these studies have added considerably to our understanding of the complexities associated with TP, the prediction for individual cases of disease expression, progression, recovery time, and mortality is still difficult, especially at the time of initial examination when an anxious owner may need a prognosis and cost estimate.

Experimentally [6], the onset of disease is highly predictable in a controlled environment (temperature, diet, exercise, and humidity), with known tick-attachment timings and sites. Clinical signs are usually not present in dogs until the fourth day after tick attachment (i.e., >72 h and an average tick size of ≥4 mm dorsal width), irrespective of tick number (e.g., 1 to 10 per animal). In a natural environment, however, the variabilities of temperature, humidity, tick age and viability, attachment timing, host immunology, physical demands of concurrent host activity, and exposure (e.g., paralysis in a hot, humid daytime or cold overnight environment) make the prediction of clinical outcome for an individual much harder [7].

In addition, many details important to the clinical management of affected dogs are anecdotal rather than evidence-based [8,9]. Veterinarians employ a wide range of drug protocols and highly variable TAS dose rates to treat cases, but the efficacy of these therapeutic approaches has not been validated [9,10,11]. We hypothesized that a study utilising a large cohort of cases from a broad geographic area may help to identify risk factors for mortality, provide evidenced-based validation for different therapeutic approaches, and ultimately inform improved clinical management of TP.

A substantial body of research in both humans and animals has described facial expressions, associated with distinct and reliable patterns of facial movements [12], which occur in states of illness and pain [13,14]. In humans, facial expressions provide an important source of information in contexts where verbal self-reports are unreliable [12]. Facial expressions communicate the intensity of the illness or painful experience and also play a role in sickness detection [15]. They have been studied in a variety of mammalian species and have been harnessed to develop pain scales for improved clinical appraisal in rodents, rabbits, horses, cattle, pigs, sheep, and dogs, with minimal time and training required for observers to gain proficiency [14,16,17].

Similarly, the visual analogue scale (VAS) has long been established in a range of human clinical and research applications as a simple, valid, and reliable technique for measuring subjective experiences [18]. VAS has been used in psychological research since the early 1900s and it has subsequently been effectively applied in a wide range of situations: rheumatic diseases, cancer, chronic pain, rhinitis, mood, appetite, dyspepsia, asthma, and disease control [19]. VAS has been shown to be highly sensitive with a discriminating capacity superior to other scales. Many studies have demonstrated the inter- and intra-rater reliability of VAS ratings, indicating that reliable measures can be obtained from untrained as well as professional personnel [19].

The use of facial expressions and VAS scores as tools for detecting illness, assessing severity, and measuring pain or discomfort has not been investigated in TP cases. The existing scoring systems used to help assess prognosis in TP cases [9] are based on specific clinical signs that may have multiple causations: tachypnoea, for example, can also be caused by hypoxaemia, anxiety, anaemia, pneumonia, etc.

The primary aims of this large prospective study were to investigate the utility of VAS scores and facial expressions as clinical prognostic tools in TP cases, and to interrogate a large body of natural case data for clinical signs that are significantly correlated to disease severity and mortality. A secondary aim was to provide evidence-based validation for different therapeutic approaches and to compare these with the results of earlier clinical studies [9,10].

## 2. Materials and Methods

### 2.1. Clinic and Veterinarian Selection

A cohort of veterinary clinics (n = 42) was recruited from areas ranging from north Queensland to Victoria to incorporate areas of high and low tick prevalence as well as to have representation from tick-endemic areas of the eastern seaboard. Clinics were selected based on their willingness to participate, their location, and their perceived standard from activities such as attendance at postgraduate/CE courses, association with university activities, clinical trial experience, or postgraduate qualifications. The following areas were represented in the trial: Queensland, including Cairns, Atherton, Toowoomba, and Brisbane; NSW, including Lismore, Casino, Port Macquarie, Newcastle, Sydney, and Eden; and Victoria, consisting of Lakes Entrance.

A representative veterinarian from each clinic agreed to attend a pre-trial tutorial and take responsibility for data recording and management of all cases selected for the trial in relation to a set completion date.

### 2.2. Case Selection

The trial involved dogs with clinical TP but without obvious concurrent acute illness, such as gastroenteritis. However, animals with concurrent chronic disease (e.g., compensated mitral valve disease) were admissible. There were no restrictions on breed, age, or gender, to enable the inclusion of a wide spectrum of clinical severities, owners, diets, home environments, remedies, etc. To accommodate practice logistics, case inclusion was restricted to those admitted during normal clinic hours and where the participating veterinarian was present on the required days to complete the questionnaire. Cases that were sent home for overnight care (due to practice policy) and returned to the clinic the following morning were included in the trial.

### 2.3. Pre-Trial Tutorial

Groups of veterinarians were instructed in a two-hour tutorial setting. The tutorial was delivered in small-group format by the investigator and the trial contact person to participating veterinarians. It covered the rationale underpinning the trial’s methodology, the aims and objectives of the trial, protocol details, and how to record trial data. Protocol instructions included detailed clinical descriptions and definitions of clinical signs to maximise descriptive accuracy. There was no formal instruction on how treatment should proceed. The concept of VAS was explained in detail along with its repeatability and published accuracy in the medical and human nursing fields [18,19,20]. The VAS scoring system (0–100, zero to severe signs) was used to capture the veterinarian’s subjective impression of each case’s overall disease severity (VAS-toxicity score), the level of respiratory dysfunction (VAS-respiratory score), and the degree of paresis/paralysis (VAS-paralysis score). The gait scoring system (1–4) [2] routinely used for clinical assessment of neuromuscular junction function (NMJ) was also reviewed in detail. Veterinarians were shown close-up photographs to define descriptions of facial expressions (anxiety, glazed eyes, fatigue) and asked to record their subjective assessment of facial expressions at the time of clinical presentation. Participating veterinarians’ understanding of the tutorial content was then tested using a one-page assessment questionnaire.

Simple cage cards were designed to allow VAS and NMJ assessments to be regularly recorded. Veterinarians were encouraged to conduct an in-house pilot trial (once back at their clinics) to practice recording VAS and NMJ scores (blinded to each other) and then to compare results for all VAS and standard NMJ assessments.

Veterinarians were asked to not crosstalk (between people and practices) about their cases and to treat and manage their cases as they normally would, with their normal selection of TAS, other pharmaceuticals, examination frequency, etc. Veterinarians were encouraged to ask questions throughout the training period and were provided with an independent contact person to assist them with any queries about data collection during the trial period. Investigator contact was limited to discussions about a case’s concurrent disease issues and its suitability for case selection.

### 2.4. Trial Design

The trial was conducted within one season, but the start date was progressive, corresponding to the tick season, as each individual clinic’s geographic latitude increased. At each location, the trial commenced after attendance at the pre-trial tutorial and continued until the clinic had completed data collection for 15 applicable cases, or reached the cut-off date, whichever came first.

For every case (anonymously classified: 1 to 15, with a practice identity code, e.g., A8) that met the trial inclusion criteria, the responsible veterinarian at each clinic was required to complete all sections of a detailed 17-page questionnaire at each examination, which recorded both subjective and objective data from the dog’s admission and hospitalisation. To help ensure consistency, clinical definitions were reproduced for reference at every examination time. The discharge questionnaire could be completed by another defined veterinarian, where illness, vacation, practice logistics, or prolonged recovery disrupted the availability of the original veterinarian for the final examination. Once finalised, veterinarians were compensated (with vouchers) in recognition of the time taken to complete the questionnaires.

The questionnaire was assessed by a focus group of independent veterinarians to ensure the wording clearly articulated the questionnaire requirements.

### 2.5. Data Collection

A detailed questionnaire was designed to record animal history, including acaricide and prevention strategies, physical examination details, clinical interventions—including TAS brand and volume used—and any concurrent compensated disease and its therapy. For each case, the VAS-toxicity score was recorded once on admission, whereas the other three VAS scores and NMJ class were recorded multiple times: at admission (designated as Day 1), 24 h later (designated as Day 2), subsequent days (during prolonged hospitalisation), and at discharge. The outcome was recorded at discharge (stage of recovery, etc.). Only disease-associated mortality was to be included in analyses of mortality; euthanasia primarily associated with financial determinants and/or treatment constraints at admission were excluded, but euthanasia due to disease severity and lack of further therapy options with disease progression were included. Owners were asked to give approval for their data to be anonymously assessed retrospectively.

### 2.6. Data Analyses

Data were entered into Microsoft Excel spreadsheets. Incomplete forms with significant omissions and forms submitted too late for the trial conclusion date were excluded. Obvious simple errors were ignored (e.g., recording the wrong year in the date) but obvious areas of poor, contradictory, or inconsistent recording of data (suggesting the veterinarians were confused by the nomenclature, design, etc.) were omitted from assessment to avoid the possibility of invalid results being generated.

The data were assessed and processed in a blinded fashion. Initial data assessment was performed by the contact person and two assistants (veterinarians), who were not associated with the survey process. In some cases of incomplete forms, if two of the three days of data were completed then these were included in those specific time-based calculations; that is, each day’s data were seen as “all dogs on Day 1”. Thus, for different factors different dog and tick data may have been used, e.g., the Days 1 and 2 data were used, but the discharge data were excluded due to timing, errors, omissions, etc. Data were assessed without access to the study veterinarians for any clarification.

Once entered into spreadsheets, the data were passed as a total data set (at different times) to two independent statisticians (who were then not contacted until data analyses were complete). Entry error was established in a randomly chosen subset of the data, which was assessed independently. The original data were kept blinded for the statisticians and the investigator during early sorting and compilation. Data that had been passed independently to the two statisticians were re-presented for qualification, further analyses, and interpretation to the investigator and also to an assistant who had been excluded from the trial performance and initial analyses. Further statistical analyses were requested after these assessments were made and ongoing interchanges continued as other issues or observations arose.

Final data analyses for the detection of predictors were based on case severity. Only highly significant (in the order of *p* < 0.01) results were chosen to be assessed for their prognostic value in a clinical setting.

Descriptive analyses were initially conducted to understand the distributions of variables. These included the calculation of summary statistics and the creation of graphical summaries for quantitative variables and frequency tables for categorical variables. Preliminary associations of explanatory variables with the binary outcome variable (mortality) were evaluated by creating box-and-whisker plots of quantitative variables and by creating contingency tables of categorical variables for each outcome.

Associations between categorical variables were tested using a chi-square test or Fisher’s exact test, as appropriate. The means of quantitative variables were compared between groups using a *t*-test (for two groups) or ANOVA (for more than two groups). Assumptions of normality and equality of variances were tested using graphical approaches. Data were transformed or non-parametric approaches were used if these assumptions were not valid.

Logistic regression analyses were conducted to evaluate associations of explanatory variables with mortality. First, univariable logistic regression analyses were conducted to test the unconditional association of explanatory variables with mortality, and then variables with a *p*-value < 0.025 at the univariable level were shortlisted for multivariable analysis. A multivariable model was created using a forward stepwise approach. Model fit was evaluated using the Hosmer and Lemeshow goodness-of-fit test.

The collected VAS data (>6000 data points) were grouped by quartile (e.g., A—up to 25; D—75 plus) for each VAS measure. Scores in each quartile for each VAS measure were summated and averaged for each day prior to analyses. NMJ scores (1 to 4) were similarly summated and averaged for each time point.

## 3. Results

Unless otherwise stated, statistical results presented here are from multivariable analyses.

### 3.1. Descriptive Data

Assessment of the tutorial feedback forms confirmed that the veterinarians had understood the material presented and the protocol details, but revealed wide differences of opinion, e.g., over what constituted a maintenance fluid-dose rate. There were less than ten questions for the contact person regarding logistical issues and one question for the investigator regarding concurrent disease and case inclusion.

Based on a subset randomly chosen to assess data entry accuracy prior to the analyses, an error rate of 0.5% was detected when 4190 data points were checked.

A total of 506 cases were originally assessed from 42 practices with an average of 12 cases per practice completed successfully. One practice had very few valid cases (Vic) while others well exceeded their case/time quota. Not all data points were available for all cases due to many reasons, including having questions omitted; conflicting and obvious errors in the interpretation of terms such as “fluid use” and “type of examination” (e.g., outside a cage versus on a table); data submitted past the closure date; and final discharge data missing. Missing data were both minor (e.g., dates incorrect) and major (e.g., no therapy data, different presumptions of fluid use—TAS dilution verses hydration).

Complete data sets for 447 cases were available. A total of 59 other cases (11.7%) were initially scored as incomplete, but at various points in the progression of analyses over time, different numbers of data points were validly available from these cases, and so varying n values appear for different analyses. The data were estimated to contain over 100,000 data points and were archived under each of the 42 practices.

**Figure 1:**
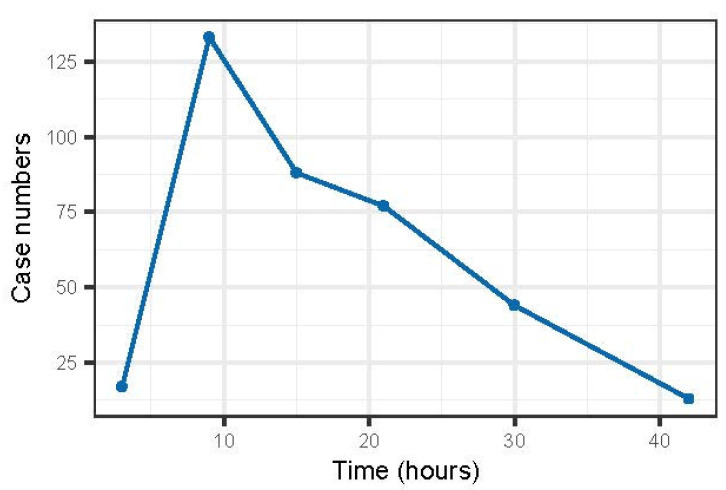
Time from initial presentation to the first sign of clinical improvement (as assessed during clinical examinations) for 360 dogs.

With regard to all completed data, a total of 416 of 447 dogs survived. The mortality rate was 6.9% (n = 31), including three dogs that were euthanised due to progressive disease. Time to recovery data (n = 360) showed early improvement in the majority of cases, but further deterioration after hospitalisation (“lag phase”) was observed in 9% (37) cases before signs of improvement were seen (Figure 1).

### 3.2. Analyses

#### 3.2.1. Animal and Tick Factors

The only variable (animal or tick) that showed any association with mortality in these dogs was age. The median age was 5.4 years (mean 4.5 years) within a range of 0.09 to 17.5 years. The median age of dogs that died was 7 years compared to 4.5 years for survivors. Dogs over 7 years old had a greater risk of death (*p* = 0.03), even after adjusting for all concurrent compensated diseases and therapies, including cardiopulmonary disease. There was an 11% increase in the probability of death from years 6 to 7 of age and a 37% increase for years 6 to 9.

No significant relationships existed between mortality rate and gender (entire or desexed; *p* = 0.89), breed (*p* = 0.86), or body weight (*p* = 0.98), although a trend was seen with heavier dogs taking longer to be discharged at the acceptable recovery level for that practice.

Brachycephalic (non-cross) dogs (n = 58) were presented to 33 clinics. A range of brachycephalic breeds (n = 12) were involved with the most common being Cavalier King Charles Spaniels and Staffordshire Bull Terriers, with Shi Tzu the next most common. Cases per clinic ranged from 1 to 6 with the average per clinic being 1.7, but the most common being one case (in 15 clinics). There was no difference (*p* = 0.86) in deterioration (5.17% vs 5.74%), time to improvement (*p* = 0.86), or time in hospital (*p* = 0.43) for these brachycephalic breeds when compared to all other breeds in this general practice population.

Table 1 outlines the tick factors that showed no association with mortality rate. Tick location was consistent with previous reports [8,14], with 81% of ticks found from the front legs forward. Owners detected 74% of ticks. Eighty percent of cases involved only one tick, 10% of cases involved two ticks, and the remaining 10% of cases involved three or more ticks. Of 455 ticks, 6.6% were dead at presentation. For dorsal width, many (34.6%, 156) measured 4 mm, 22% measured 3 mm, and 22% measured 5 mm, but mean tick size did not vary between geographical areas. There were no associations between VAS scores and the number or size of ticks present.

#### 3.2.2. Predictors of Outcome—NMJ and VAS Scores

**Figure 2:**
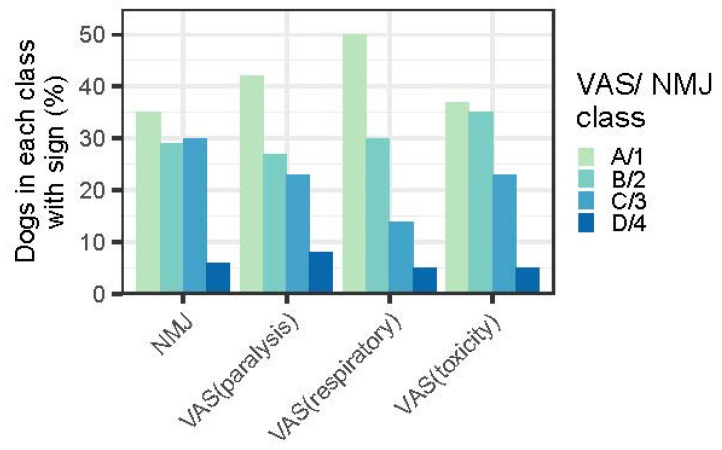
Prevalence of disease severity in cases of TP as assessed using three VAS scores (toxicity, respiratory, and paralysis) and the original NMJ scoring system (1–4) [2]. VAS scores were grouped by quartile (0–25—A; 25–50—B; 50–75—C; and 75–100—D) prior to analysis.

Figure 2 shows the distribution of disease severity as measured by the four assessment methods. There was some variability between the assessment methods for the three lower quartiles, but the upper quartile was more consistently scored by all four methods. The VAS-respiratory score was the most effective scoring system for predicting mortality, clearly differentiating the four categories of severity.

From over 6000 NMJ and VAS scores that were recorded, univariable analysis showed that the VAS-respiratory scores were most predictive of a poor prognosis, with the upper quartile (D) having a 36.4% cumulative mortality and an OR of 30.3, relative to the lower quartile (Table 2). The VAS-paralysis score had similar predictive results to the NMJ score at all score levels, e.g., VAS-paralysis score D had an OR of 15.5 and a mortality rate of 30.3% whereas the NMJ 4 score had an OR of 14 and a mortality rate of 32.0% (Table 2). The VAS-toxicity assessment had highly significant (*p* < 0.0001) OR scores for the D quartile (19.9), with a D group mortality rate of 39.1% (*p* < 0.001).

There were minimal differences in mortality rates between VAS scores A and B and NMJ 1 and 2, but probabilities increased substantially to VAS C and NMJ 3 and then exponentially to VAS D and NMJ 4 i.e., A = B, <C, <<D. This trend applied to all four averaged assessment scores for both mortality rates and OR values (Table 2).

The most reliable of the four classifications for predicting mortality were the VAS-respiratory score (*p* = 0.005) and the NMJ score (*p* = 0.003). The OR values for both these assessments increased from the lower to the upper quartile; for the VAS-respiratory score they were 1, 4.1, 7.2, and 19.3 (*p* = 0.005) for the ascending quartiles, and for the NMJ score they were 1, 0.2, 0.8, 4.26 (*p* = 0.003). The conjoined OR for the most severe categories of both assessments (i.e., upper quartile D for the VAS-respiratory score and class 4 NMJ) was 16.6. Thus, a D4 classification had approximately a 16-fold-increased risk of death.

Analysis of the VAS-toxicity score assessed at admission by veterinarians produced OR values (A to D) of 1, 15.6, 15.1, and 79.3 with associated mortalities of 0.7, 10.3, 10.0 and 37.8, respectively.

Higher clinical scores (C + D > A; 3 + 4 > 1) of the three VAS assessments (toxicity *p* < 0.0001, OR > 3.1; respiratory *p* < 0.004, OR > 2.0; paralysis *p* < 0.0001, OR 3.9) and NMJ scores (*p* < 0.0001, OR 3) were also all associated with longer recovery times.

#### 3.2.3. Predictors of Outcome—Specific Clinical Signs

**Figure 3:**
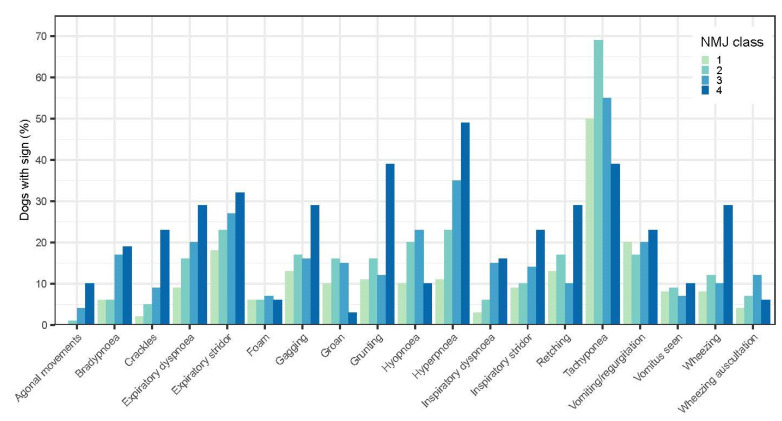
The percentage of dogs in each NMJ class [2] displaying each of 19 defined clinical signs. No predictable pattern of clinical signs could be related to disease severity, as measured by NMJ scoring.

**Figure 4:**
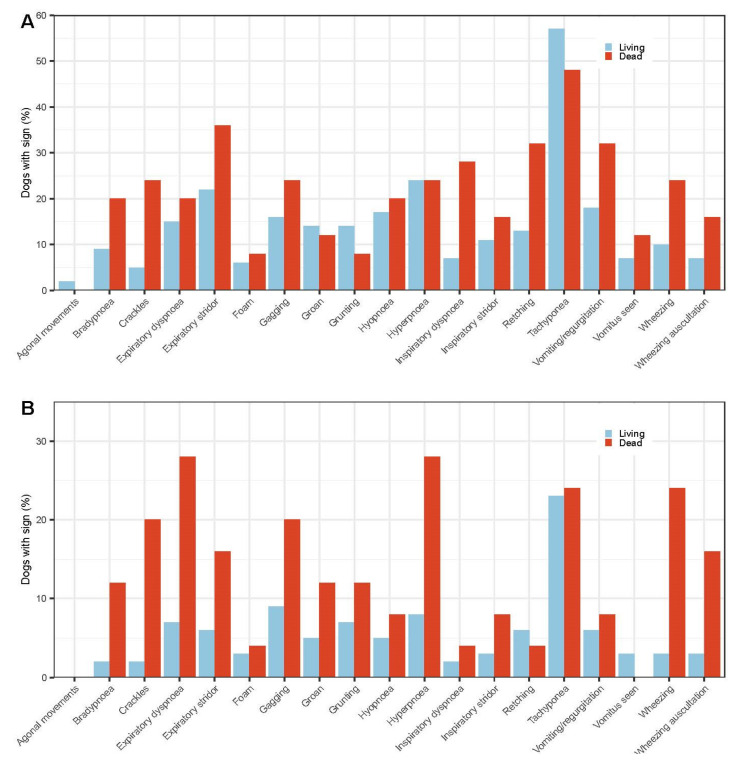
The percentage of dogs with defined respiratory and other signs in relation to mortality (**A**) on day 1 and (**B**) on day 2. The most highly significant signs in relation to mortality were crackles and inspiratory dyspnoea on day 1 and wheeze and expiratory dyspnoea on Day 2.

Dogs that died had an average of 3.6 ± 2.4 clinical signs (with each additional sign increasing the risk of mortality by 23%), whereas those that lived had an average of 2.5 ± 2.0 signs. However, when each of the pre-defined clinical signs (e.g., breathing pattern, polypnoea) were assessed against NMJ scores (Figure 3), no progressive prognostic pattern could be detected that would aid case predictability. When each clinical sign was assessed in relation to time (i.e., Day 1 and Day 2), no consistent pattern (Figure 4A,B) could be seen that would verify the use of the respiratory score previously used in general practice and in other tick studies [21].

The presence of inspiratory dyspnoea (*p* = 0.002, OR 5.3, CI 1.9–13.0) and crackles (*p* = 0.003, OR 5.7, CI 1.9–15.0) on Day 1 were the most significant prognostic indicators of mortality. Mortality was associated with 21.4% of cases that presented with crackles compared to 4.6% of cases that did not so present, and with 19.4% of cases presenting with inspiratory dyspnoea compared to 4.4% of cases that did not. Dogs with either of these specific signs had a five-fold-increased risk of death.

On Day 2, the presence of expiratory dyspnoea (15% dogs; *p* = 0.0005, OR 4.2, CI 1.5–11.6) and an expiratory wheeze (*p* = 0.0001, OR 9.1, CI 2.9–28.1) were the most significant prognostic indicators of mortality, but the presence of inspiratory or expiratory stridor was not associated with mortality (*p* = 0.19 and *p* = 0.07, respectively).

Retching was associated with both a higher probability of mortality (14% dogs; *p* = 0.02, OR 3.1, CI 1.2–7.3) and longer hospitalisation times (*p* = 0.02, OR 0.38, CI 0.2–0.9), while grunting (*p* = 0.05, OR 1.7, CI 0.7–8.7), and gagging (*p* = 0.001, OR.1.92, CI 0.7–6.6) were associated with delayed recovery.

#### 3.2.4. Predictors of Outcome—Facial Expressions of Distress

The results of the analyses of subjective observations of defined facial expressions were profound; anxiety (*p* = 0.003, OR 5; mortality of 18.9% compared to non-anxiety mortality of 4.5%), glazed eyes (*p* = 0.015, OR 5.5; mortality of 22.2% compared to 5.0% non-glazed), and fatigue (*p* < 0.001, OR 10.4; mortality of 31.8% compared to 4.3% non-fatigued) were all highly predictive of death and the OR approximately doubled (*p* = 0.001) for cases with greater than one facial sign (one sign *p* < 0.001, OR 4.9). Increased risk of death was associated with additional facial signs. These facial signs are presumably caused by various aspects of disease expression, and when present they indicate a risk of mortality increased by 5- to 10-fold and are a reliable indicator of a poor prognosis.

#### 3.2.5. Geographic Variation in Clinical Disease

Data from the 42 sites were grouped into four areas—northern coastal, northern inland, southern coastal, and southern inland—using the Great Dividing Range and the Queensland–New South Wales border to classify areas as coastal or inland and north or south, respectively. Analyses to determine whether clinical signs varied between locations demonstrated that southern coastal regions had significantly higher VAS-toxicity scores (*p* = 0.0068; OR 1.6; CI 1.0–2.6) compared to the other areas, with 7.2% of cases scored as VAS toxic class D and 26% as class C. The frequency of defined respiratory signs also varied between areas, with cases from southern inland areas having more respiratory symptoms than those from other areas (tachypnoea *p* = 0.003; hyperpnoea *p* < 0.001; bradypnoea *p* < 0.0001; expiratory stridor *p* = 0.004; gagging *p* = 0.04). However, overall disease severity (*p* = 0.4), time to improvement (*p* = 0.2), and mortality (*p* = 0.55) did not vary between these areas.

#### 3.2.6. Pharmaceutical Product Use and Supportive Treatments

The only product that was positively associated with mortality was TAS (*p* < 0.001). Mortality rate was not affected by the brand (n = 4) of TAS used (*p* = 0.4), the dose/volume administered (*p* = 0.9), or the time of TAS administration (*p* = 0.5 to 0.9). Mean TAS dosage in dogs that died versus those that survived was 14.1 mL (+/−7.6) and 14.3 mL (+/−8.4) (*p* = 0.90), and 1.1 (+/−0.5) and 1.0 (+/−0.5) mL/kg (*p* = 0.73), respectively. TAS was used as the only therapy in 34 mild cases (VAS-respiratory score A–B; NMJ 1–2) and all of these survived. In a cohort of 407 dogs, TAS use plus one other drug was associated with a mortality of 6.1% (n = 25). Three mild cases (VAS-respiratory score A, NMJ 1) had no TAS and all of these survived.

In clinical cases assessed by NMJ (1–3) or VAS (A–C), antibiotic use (but not one specific class) was negatively correlated with mortality (*p* = 0.018, OR 3.08, CI 1.23–7.42), as more dogs receiving antibiotics died compared with those that did not receive antibiotics. However, in the most severe classification of the VAS-respiratory score, the use of antibiotics was positively correlated with outcome (*p* = 0.018, OR 3.1, CI 1.2–9.4). For all four assessment methods, the more severe the case the more likely antibiotics were used (NMJ 4 > 1 and VAS D > A; 2.7 to 10 times more likely; *p* = 0.02 to <0.001).

Diuretic use (in 13.1% of cases) was also negatively correlated with mortality (*p* = 0.02; OR 3.29; CI 1.2–8.02). Of the 51 cases so treated, 44 recovered and seven died (13.7%). In the remaining cases (with a similar drug-use profile) that were not treated with diuretics (n = 39), only 5% died. However, as with antibiotics, diuretics were more likely to be used in the most severe cases (e.g., VAS-respiratory score D, 5.4 times more than A, *p* = 0.0014; and NMJ score 4, 4.7 times more than NMJ score 1, *p* = 0.0071).

Many different pharmaceutical products were used on the 506 dogs. In general, more classes of products were used as severity increased. Risk of death was associated with multiple product use, increasing by 23% with each additional drug class (*p* = 0.003, OR 1.23, CI = 1.04–1.43). Although other drug types that were used (acepromazine, atropine, steroids, antihistamines, antiemetics, and S8 anti-anxiety and sedation drugs) showed no significant associations with mortality (*p* values from 0.1 to 0.8), the use of antihistamines (*p* < 0.0001), antiemetics (0.015), or diuretics (*p* = 0.001) were all associated with longer hospitalisation times. Analysis of individual cases showed that the use of antiemetics did not necessarily follow clinical indications: antiemetics were used in only 40% of dogs where vomit was observed and were used in 6% of dogs where vomit was not observed.

Similarly, associations with delayed recovery were found for supportive care such as clipping (0.02, OR 1.7, CI 1.1–2.7), eye care (*p* = 0.014, OR 1.7, CI 0.36–1.55), bladder care (*p* = 0.001, OR 3.7, CI 0.34–7.8), and frequency of examination (*p* = 0.0001, OR 0.55, CI 0.19–1.48).

Fluid-use data were hard to assess because the protocol format did not clearly define the term, leading to a poor distinction between fluids used for dehydration treatment, for maintenance, for TAS dilution, or for multiple purposes. Of the 493 dogs assessed, 12% were considered to be dehydrated based on varying combinations of skin turgor, packed cell volume, and total plasma protein, but the degree of dehydration was not recorded. The rate of intravenous fluid administration was noted in 82 cases and varied widely: 16% were administered between one-third and half of normal maintenance rates; 43% were administered between 1.5- and 3-times normal maintenance rates and the remainder were delivered at maintenance rates. Fluid therapy to correct dehydration (n = 59) significantly improved recovery time (98% dogs; *p* = 0.005; OR 7, CI 1.5–126).

Manual ventilation was used in only 5 of 506 cases, which were too few to analyse. None of the clinics involved had access to intensive care facilities.

Of the cases that were hospitalised, 50% had not received tick preventative products prior to hospitalisation. Acaricide use after hospital admission (n = 253) resulted in a 2.2-times (OR) advantage with respect to hospitalisation time (*p* = 0.027; CI 1.0–3.9).

## 4. Discussion

This study demonstrated the accuracy and validity of using VAS-respiratory and VAS-paralysis scores in combination with specific clinical signs for assessing TP case prognosis and consequently informing clinical management. The prognostic accuracy of the VAS scoring tools was not surprising. The VAS-respiratory score provides an estimate of overall respiratory distress, which increases with increasing severity of TP due to varying combinations of pulmonary and alveolar ventilatory dysfunction. Ultimately, this respiratory dysfunction is the cause of death [22]. The VAS-paralysis score produced similar results to the original neuromuscular scoring system [2], which also employed highly repeatable scoring criteria that reliably predicated outcome. To date, the most widely used tool in clinical and research assessments of TP severity is a respiratory score based on individual respiratory signs [21], but our data found no correlation between any of the well-defined respiratory signs and prognosis. This result can be explained, at least partly, by the fact that many signs can have several causes, such as fear, pain, stress, and hypoxaemia. Importantly, the presence of inspiratory dyspnoea and/or crackles (perhaps reflecting alveolar lesions) on the admission of dogs with TP were reliably associated with higher mortality; similarly, the progression to expiratory dyspnoea and expiratory wheeze (possibly reflecting increased airway resistance, especially if grunting was present) within 24 h of hospital admission was reliably associated with higher mortality.

The presence of retching/vomiting was predictive of a prolonged recovery and a poor prognosis. Interestingly, clinicians’ subjective assessments of facial expressions on admission to hospital were found to be profoundly predictive of a poor prognosis. This finding adds to a large body of human and animal research that demonstrates the utility of facial expressions to inform clinical appraisal [14]. In dogs, tight facial muscles, especially around the eyes, conveys anxiety with or without pain; a vacant or glazed stare conveys profound fatigue or pain [23]. These states, when evident at admission in conjunction with other specific clinical signs, justify early intensive care therapy. Further research to develop these perceptual cues into clinical scales for improved assessment in TP cases is warranted.

In this study, dog age was highly correlated to mortality after age 7, with mortality increasing significantly with incrementally increasing age. This finding concurs with previous clinical studies [9]. Why age should be so significant is not known, but it may simply reflect reduced organ reserves with ageing, such as reduced lung capacity.

Although there were no significant differences between geographic areas for disease severity or mortality, both respiratory signs and overall toxicity (as measured by VAS-toxicity scores) did vary between broad geographic areas. It is reasonable to assume—with 506 cases involving many breeds and spread over such a large geographic area—that genetic variations in dog immune responses to tick toxins are probably not playing a role in the observed variation in clinical signs and toxicity. Rather, this finding suggests there may be toxin-profile variations between regions, which is supported by previous research [24] that has demonstrated genetic differences in *I. holocyclus* from different geographic regions. Some ticks have been shown to not induce any toxicity [25]. Furthermore, work by the tick toxin research group [26] has observed many toxin types from tick saliva, some of which were TAS sensitive and some of which were not, perhaps revealing why TAS may not be as effective as expected in some cases. Variability in toxin-supply rate from different tick sites has also been proposed as a mechanism to explain research findings demonstrating that large and small ticks and high and low tick numbers can all produce mild or severe disease [27,28].

These data did not demonstrate any association between the severity of clinical signs or overall mortality and the size and number of ticks found, concurring with previous findings [9] and supporting the hypothesis that multiple factors underpin disease severity and mortality. These likely include toxin-profile variation that may produce different clinical signs, as well as toxin-supply rate, speed of toxin transport and binding, and innate host immunity/susceptibility. Anecdotal reports from practitioners in ‘hot seasons’, where cases deteriorate rapidly, and ‘mild seasons’ when less severe signs are observed even with large, engorged ticks (≥9 mm), further supports this hypothesis.

Most cases were mild to moderate (A1/B2; 80% of cases assessed by VAS respiratory score) and could be expected to improve within 12 h of admission, while others took up to 36 h or longer to improve. Not surprisingly, supportive care (clipping, eye, and bladder care, and frequent examination) was associated with delayed recovery, but all these factors were a reflection of case severity and therefore the need for such care. A small percentage of cases (e.g., D4 cases and those that died) may have benefited from early intensive care with the appropriate facilities for mechanical ventilation. The only drug that showed a significant association with improved clinical outcomes across all cases was TAS; this was observed despite differences in product brand and a wide variation in administered doses and case management techniques.

Antibiotic use was also significantly associated with mortality, but the association was only positive in the most severe cases (upper quartile). This result is not surprising as alveolar oedema and/or infection are often associated with severe paralysis [22], especially where crackles are present. None of the other drug classes used by practitioners in this study correlated positively with either mortality or improved recovery time, corroborating the results of previous clinical studies [9]. However, increasing the number of drug classes used did correlate with delayed recovery, longer hospitalisation times, and higher mortality. These findings are likely a reflection of practitioner frustration, with poor recovery leading to an increased drug class usage in an attempt to maximise the chance of a positive outcome (despite a lack of research evidence for the benefits of such drugs). This would explain the negative correlation found for antibiotic use in less severe cases (lower 3 VAS quartiles), where death was occurring from causes other than infection. Similarly, antiemetic use was negatively associated with mortality. However, meaningful interpretation of this result is problematic because, when analysed in conjunction with clinical data, antiemetics were underused when indicated and overused when not indicated, perhaps reflecting the absence of empirical data to support the use of such therapy for TP cases. Further controlled clinical studies to investigate the benefits of these and other pharmaceuticals across the spectrum of disease severity are warranted.

Acaricide use to help remove all hidden ticks and rehydration therapy were both correlated with a marked clinical benefit in this study. Routine acaricide use for all tick cases is a good practice recommendation that is supported by our data. However, the same cannot be said for the administration of fluid therapy in cases that are not clinically dehydrated. With hindsight, fluid-therapy reporting in this study should have been more detailed and nuanced. The fluid-therapy data collected was not detailed enough to draw meaningful conclusions about the observed benefits in cases other than those that were clinically dehydrated. However, other published research indicates that caution should be exercised when administering fluids to dogs with TP. Findings that over 30% of TP cases (and not just the most severe cases) show evidence of pulmonary vein dilation [29] and around half of ventilated tick paralysis dogs that die have pulmonary oedema on post-mortem [3,22] suggest that care should be taken to not overhydrate these animals. Our data showing the prognostic power of crackles to predict mortality reinforces recommendations for caution in over-administering fluids. Defining the cause of any crackles, for example by radiography, would seem sensible as pneumonic dogs need to be well hydrated whereas oedematous dogs need to be hydrated with caution. The fact that 43% of fluid-dose rates used in this study were above maintenance but only 12% of dogs were identified as dehydrated suggests that some cases may be overhydrated.

This study has also provided empirical evidence for treatment priorities in TP. Acaricide use on admission to hospital, TAS administration, rehydration, and antibiotic use (in severe cases) should be the focus of drug therapy in general TP cases, with fluid rates carefully correlated to individual needs. Diuretics and antiemetics should be used more carefully. The use of either drug type in TP cases needs to be urgently studied.

## 5. Conclusions

This study represents the largest clinical dataset of TP cases managed in general practices in Australia, and as such provides valuable guidance for practitioners dealing with canine TP. VAS scores, facial features on admission to hospital, specific respiratory signs on Day 1 and Day 2, and negative prognostic indicators such as vomiting/retching and patient age, can be utilised by veterinarians for making timely decisions to prioritise more intensive management, such as timely mechanical ventilation or referral.

## Figures and Tables

**Table 1:** Factors that had no effect on mortality based on the unconditional logistic regression analyses of these variables for mortality.

Factor	*p*-Value
Tick number	0.89
Tick size	0.97
Whether ticks were plucked	0.46
Whether diagnosis was by presence of crater or tick	0.63
Whether ticks were alive or dead	0.32
Adult or non-adult stage of tick	0.66
Whether tick was located on presentation at clinics or removed at home prior to presentation	0.18
Whether tick was detected by owner or by clinic	0.46
Cardiopulmonary disease	0.37
Other concurrent disease	0.75
Concurrent (non-tick) drug therapy	0.77

**Table 2:** Odds ratio (OR) and mortality rates from univariable analyses for all VAS and NMJ scores.

Scores	OR—A1 (Mortality %)	OR—B2 (Mortality %)	OR—C3 (Mortality %)	OR—D4 (Mortality %)
VAS-_TOXICITY_	1 (3.1)	0.6 (2)	2.6 (7.8)	19.9 ** (39.1) *
NMJ	1 (3.2)	0.5 (1.6)	2.5 (7.6)	14 (32)
VAS-_PARALYSIS_	1 (2.7)	0.6 (1.7)	2.7 (7)	15.5 (30.3)
VAS-_RESPIRATORY_	1 (1.9)	2.6 (4.7)	6.6 (11.1)	30.3 ** (36.4) *

* *p* < 0.001; ** *p* < 0.0001.

## Data Availability

The data presented in this study are available on request from the corresponding author. The data are not publicly available due to Merial’s standard commercial in confidence protocols.
